# Non-Additive Increases in Sediment Stability Are Generated by Macroinvertebrate Species Interactions in Laboratory Streams

**DOI:** 10.1371/journal.pone.0103417

**Published:** 2014-08-07

**Authors:** Lindsey K. Albertson, Bradley J. Cardinale, Leonard S. Sklar

**Affiliations:** 1 Department of Ecology, Evolution, and Marine Biology, University of California Santa Barbara, Santa Barbara, CA, United States of America; 2 School of Natural Resources and Environment, University of Michigan, Ann Arbor, MI, United States of America; 3 Department of Earth & Climate Sciences, San Francisco State University, San Francisco, CA, United States of America; Stanford University, United States of America

## Abstract

Previous studies have shown that biological structures such as plant roots can have large impacts on landscape morphodynamics, and that physical models that do not incorporate biology can generate qualitatively incorrect predictions of sediment transport. However, work to date has focused almost entirely on the impacts of single, usually dominant, species. Here we ask whether multiple, coexisting species of hydropsychid caddisfly larvae have different impacts on sediment mobility compared to single-species systems due to competitive interactions and niche differences. We manipulated the presence of two common species of net-spinning caddisfly (*Ceratopsyche oslari, Arctopsyche californica*) in laboratory mesocosms and measured how their silk filtration nets influence the critical shear stress required to initiate sediment grain motion when they were in monoculture versus polyculture. We found that critical shear stress increases non-additively in polycultures where species were allowed to interact. Critical shear stress was 26% higher in multi-species assemblages compared to the average single-species monoculture, and 21% greater than levels of stability achieved by the species having the largest impact on sediment motion in monoculture. Supplementary behavioral experiments suggest the non-additive increase in critical shear stress may have occurred as competition among species led to shifts in the spatial distribution of the two populations and complementary habitat use. To explore the implications of these results for field conditions, we used results from the laboratory study to parameterize a common model of sediment transport. We then used this model to estimate potential bed movement in a natural stream for which we had measurements of channel geometry, grain size, and daily discharge. Although this extrapolation is speculative, it illustrates that multi-species impacts could be sufficiently large to reduce bedload sediment flux over annual time scales in streams where multiple species of caddisfly are present.

## Introduction

In most fields of natural science, the historical view has been that the abundance, distribution, and biological diversity of organisms are controlled by spatial and temporal variation in the abiotic environment [1], [2]. However, several emerging paradigms in ecology, evolution, and biogeography have increasingly emphasized that the abundance, distribution, and diversity of organisms are not simply byproducts of the abiotic environment. Instead, organisms can directly control the physical formation of habitats [3], the fluxes of elements that regulate Earth’s biogeochemical cycles [4], [5], and the efficiency by which limiting resources are captured and converted into new biomass [6], [7].

In river systems, for example, physical processes such as flooding, drought, and sediment erosion have long been thought to play key roles in ecosystem dynamics [8], [9]. However, a growing number of case studies have shown that biological structures (e.g., plant roots on riverbanks) must be incorporated into physical process models to predict the evolution of channel morphology and downstream movement of water, sediment, and nutrients [10]–[12]. Research indicates that different types of organisms such as plants vary in traits (e.g. rooting depth) in ways that can differentially influence transport processes like bank erosion [13]. But the majority of these studies have quantified the impact on a transport process of a single, dominant organism and assumed that the species exists as a monoculture. Only a few studies have considered that natural habitats consist of a diverse assemblage of interacting species that must use their environment differently in space or time to coexist [14], [15]. If coexisting species use their environment differently, and the biological structures created by these species impact processes that influence sediment motion, then accounting for interactions among species will be essential to developing better predictive models of sediment transport.

Here we investigate how competitive interactions among macroinvertebrate species impact the threshold shear stress required to initiate motion of riverbed sediments (commonly called “critical” shear stress). The transition from a stable bed to a bed that is moving is a fundamental attribute of river dynamics. The shear stress required to cross that threshold directly affects the long-term sediment flux, the temporal distribution of transport, and characteristics of channel morphology such as width and the spacing of bars [16], [17]. For a given sediment grain size, measurements of critical shear stress vary by a factor of two or more for hydraulically rough conditions [18], suggesting the need for more precise estimation. Some have suggested increased precision may come from including biological controls on sediment transport conditions [19], [20], though this proposition has been infrequently tested.

**Figure 1:**
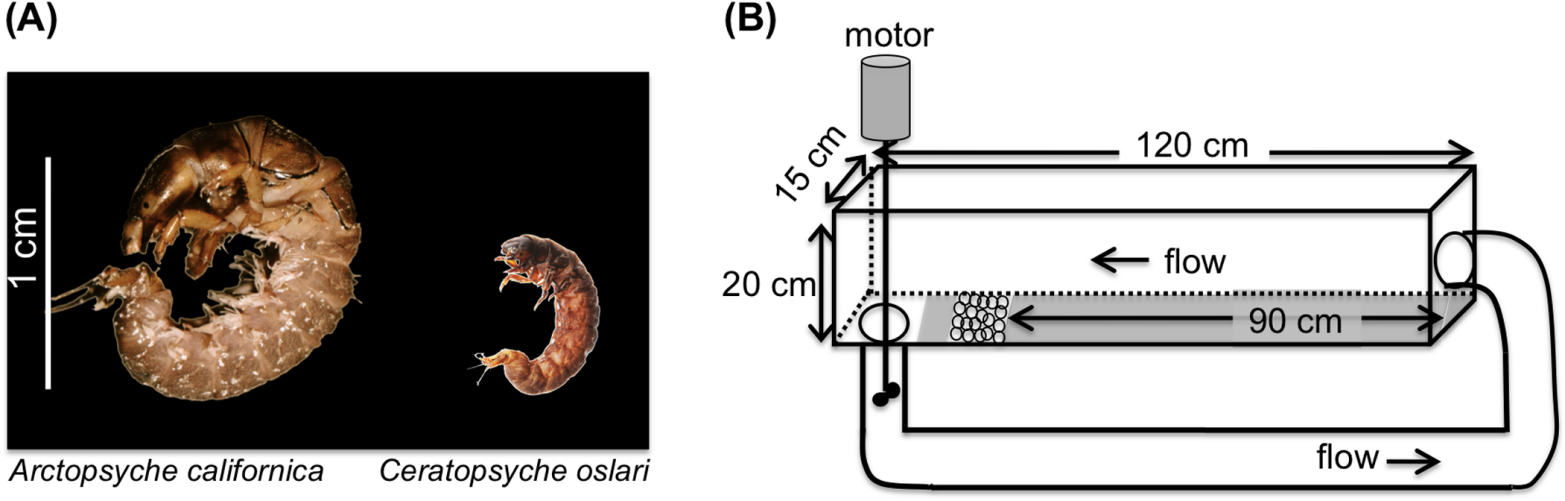
Experimental methods. (A) The two caddisfly (Trichoptera: Hydropsychidae) species used in the experiment. These caddisflies have a fully aquatic larval life-stage during which time individuals spin nets in the benthic substrate to filter feed. *Arctopsyche* is, on average, twice as long and 7 times as heavy as *Ceratopsyche* (Figure S1). (B)The flume mesocosms used in the experiment (1.2-m long × 0.15-m wide × 0.20-m deep). A motor attached to a shaft and propeller recirculates water through the flume and over the sediment patch. Grey shading along the flume bottom represents the plexiglass false-bottom leading up to and behind the working patch of sediments.

The species that we studied were hydropsychid caddisflies (Trichoptera:Hydropsychidae), a group of insects that live in streams (Figure 1A). As larvae, the hydropsychid family of caddisflies live in the benthic habitat where they construct nets composed of silk threads across the interstitial spaces between sediment particles to capture food. They often occur in high densities (>10^3^ m^−2^) in riffle habitats [21], [22], where bed sediments typically remain immobile until flow depth approaches bank-full. Previous studies have shown that these silk nets can bind sediments together, thereby increasing the critical shear stress required for incipient motion during high discharge events [22]–[24]. Although hydropsychid caddisflies are a geographically diverse group [25], no studies to date have explicitly monitored whether caddisflies in streams that contain multiple species have the same impact on sediment mobility as those that contain just one species. Given their life-history characteristics, there are several *a priori* reasons to hypothesize that multi-species assemblages might impact incipient motion differently than single species assemblages. First, body size differs considerably between species, leading to differences in the size of rocks, pore spaces, and flow velocities in which they construct nets [26], [27]. Second, when densities are high, caddisflies are aggressive, and compete intensely for suitable space to construct their nets [28], [29]. Taken together, the potential exists for competition to drive differential use of benthic habitat by species that differ in body size.

To determine whether competitive interactions cause multi-species communities to have different impacts on the threshold of sediment motion compared to single species, we manipulated caddisfly richness in sediment patches that were subjected to simulated floods in laboratory mesocosms. We compared the threshold of incipient sediment motion to address two questions: (i) do caddisfly monocultures increase the threshold of incipient motion above that of controls with no caddisflies, and (ii) do polycultures of caddisflies influence incipient motion differently from what could be predicted from their impacts in monoculture? We used behavioral trials to determine if interactions between species in polyculture could account for changes in net arrangement in a way that might alter sediment mobility. Finally, we used our data to parameterize a common model of sediment transport to explore whether the changes in critical shear stress documented in the laboratory experiment would be sufficiently large to translate into meaningful effects on bedload transport in a real stream.

## Methods

### Study organisms

Insects in the net-spinning family Hydropsychidae are geographically widespread and often occur in polyculture [30]. In 1254 streams sampled as part of the United States Environmental Protection Agency’s Wadeable Streams Assessment, hydropsychid caddisflies were present in 705 streams, and of those 705 streams, 56% were inhabited by two or more species. In the Sierra Nevada Mountains, where we conducted this experiment, two common species – *Arctopsyche californica* and *Ceratopsyche oslari* (Figure 1A) – are typically abundant during late spring and through the summer during the season of peak discharge for this snowmelt-fed system [31], [32]. The particular populations used in this experiment were collected as 3^rd^–5^th^ instars from McGee (lat 37°35′N, long 118°47′W) and Swauger (lat 38°16′N, long 119°17′W) Creeks near Mammoth Lakes, CA, USA.

### Experimental mesocosms

To experimentally recreate the interactions of caddisfly larvae and bed sediments under controlled flow conditions, we used a set of four clear plexiglass flumes (1.2-m long × 0.15-m wide × 0.20-m deep) housed at the Sierra Nevada Aquatic Research Laboratory (SNARL). Each flume recirculated water by a direct current (DC) motor (Bodine) attached to a stainless steel shaft with a 10-cm diameter propeller that was regulated by a speed control console (Minarik) (Figure 1B). Water was pumped from nearby Convict Creek (no specific permit required) into the wet laboratory at SNARL, maintained at ambient stream temperature (17–19°C), and the flumes were refilled with fresh, filtered stream water at the beginning of each replicate.

We used a reach of Convict Creek that flows through SNARL as a prototype for the experimental conditions in the flumes. To simulate bed surface conditions, we installed a 55 mm thick patch of sediments in a 0.15-m × 0.10-m recessed test section in each flume such that the mid-point of the surface grains was flush with a plexiglass flume bottom. The sediment patch was located 90 cm downstream from the flume entrance to allow full flow acceleration. The bed of Convict Creek has a coarse surface layer, as is common in gravel bedded streams [33]. Accordingly, we used a single layer of 22 mm (intermediate axis) rounded grains at the surface, underlain by two sequentially finer subsurface layers of 12 mm and 5 mm grains, respectively; each subsurface layer was 2 grain diameters thick. Our goal for this experiment was to replicate a subset of field conditions where a gradient of pore space size with depth might create niche opportunities for the different study species due to their differences in body size (*Ceratopsyche* length = 7.8±0.13 mm and *Arctopsyche* length = 17.6±0.18 mm; Figure S1). Although the two species were significantly different in body size (Figure S1), we measured body mass toward the beginning and end of the experiment (July 7 and August 2) and found that mass was relatively consistent for individuals within a species (*Ceratopsyche* mass = 0.004 g in July and 0.003 g in August and *Arctopsyche* mass = 0.015 g in July and 0.018 g in August). The grains were placed by hand into the sediment patches to maintain a loose packing characteristic of recently deposited sediments in a zone of active bedload transport.

We chose to work at the scale of these flumes because they are sufficiently large to capture caddisfly preferences for net-building locations within the benthic substrate and to accurately measure fluid shear stresses, yet they are small enough to feasibly conduct a well replicated study. As described in detail below, we can scale our results from the flumes to field conditions with standard non-dimensional ratios routinely used in laboratory modeling of hydraulic and sediment transport processes [34]. The controlled physical conditions in the flumes are easily reproduced across individual flumes and replicates, ensuring reliable comparisons of the impacts of mono- and polycultures of caddisfly larvae to control flumes that have no larvae, which allows us to detect biologically-driven changes in incipient grain motion relative to control flumes that have the same physical properties.

### Experimental design

The experiment was performed as a complete randomized block design in which each of four caddisfly treatments was randomly assigned to one of four flumes during temporal blocks [35]. The experiment was replicated in eight blocks that were run on successive dates between 1 July and 30 August 2009. The four caddisfly treatments were: *(i)* a control with no caddisflies, *(ii)* a monoculture of *Arctopsyche*, *(iii)* a monoculture of *Ceratopsyche*, and *(iv)* a 50∶50 polyculture of *Arctopsyche* and *Ceratopsyche*.

At the beginning of each block, the sediment patches were populated by gently placing caddisfly larvae in the water just upstream and allowing them to drift into the sediments. Caddisfly individuals from both species had been collected by hand within the previous 12 hours, gently placed in buckets with stream water and mesh netting that provided structures on which they could crawl, and held at ambient stream temperature. The target density in the experiment was 2000 m^−2^, which is in the range of densities commonly found in Convict Creek [31] and similar to densities in the 1000 s m^−2^ reported for other gravel-bedded rivers [22], [36]. Because we did not have prior data to suggest how and at what densities the caddisflies might colonize the sediments in the laboratory flumes, we achieved our target density by first estimating the carrying capacity for both species in the flumes (Figure S1) prior to the experiment. We then selected a given percentage of that carrying capacity for the initial additions of larvae, which represented a tradeoff between the effort required to collect thousands of individuals and ensuring that density was high enough for frequent interactions among individuals to occur. Based on initial additions of larvae to the flumes during the experiment, the average number of individual caddisflies (N = 8 replicates ±1 SEM) that ultimately settled in the sediment patches of monocultures was 23±5 and 37±9 per sediment patch with bed area of 0.015 m^−2^ (Figure S2) for *Arctopsyche* and *Ceratopsyche*, respectively, which was equivalent to 1,530±130 m^−2^ for *Arctopsyche* and 2,460±230 m^−2^ for *Ceratopsyche* (Figure S2). These final larval densities were 69% and 40% of the maximum possible density for *Arctopsyche* and *Ceratopsyche*, respectively (Figure S1). In polyculture, final settling density was intermediate between the two monoculture treatments, averaging 29±3 per patch, which was equivalent to 1,933±200 individuals m^−2^. Despite some variation in the density across species and replicates, the range of densities achieved was narrow and had no statistically significant effect on the results (described below; Table S2). After drifting into the sediment patch, larvae were given a 4-day colonization period, which allowed enough time for them to both search for a suitable location to settle and to build a complete silk net (Figure 1B). During this time, the larvae were fed Hikari pulverized algae wafers >50 particles/mL, which is a supply of food that has been shown to be sufficient for caddisfly larvae in previous studies that investigated resource capture and silk net filtration [6], [29]. When individuals drifted past the sediment patch, they were recirculated through the flume until settling occurred.

### Simulated flood

After the colonization period, we simulated the rising limb of a flood event to test whether the threshold shear stress for sediment motion was different in sediment patches containing *(i)* caddisfly monocultures versus the controls with no caddisflies and *(ii)* one versus both caddisfly species. To do this, we gradually increased flow velocity by incrementing motor dial speed until we visually observed at least one rock moving out of the test patch into a 1 mm mesh net located 5 cm downstream of the patch. The criterion of first movement has been widely used in previous laboratory studies of initial sediment motion [18]. After incipient grain motion was determined, discharge was increased in consistent increments and held for 1 minute at each increment until the flume motor was at maximum output. For each incremental increase, grains and caddisflies that moved out of the patch were caught in the downstream net, collected, preserved in 75% ethanol, and counted later.

To estimate the bed shear stress at initial motion, we constructed a calibration curve relating motor dial speed to shear stress calculated from measurements of vertical velocity profiles. It was not feasible to measure velocity profiles when caddisflies and their nets were present during the actual experiment because the bed geometry and thus the local shear stress changed in our flumes once grain motion began. To overcome this problem, we used sediment patches with a surface layer identical to that in the caddisfly treatments (*D_50_* = 22 mm) in which the grains were cemented together so that we could obtain stable measurements of velocity and shear stresses at and above the critical value without grains moving. While constructing the calibration curve, the net used to catch moving sediment grains and caddisflies during the experiment was present and located in the same position as in the experiment. We measured velocity profiles (Figure S3) above the center of each patch using an Acoustic Doppler Velocimeter (Nortek) at increasing motor dial speeds and calculated shear stress (*τ_b_*) as 
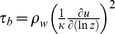
 where *ρ_w_* is the fluid density, κ is the von Karman constant (κ = 0.4), *u* is velocity, *z* is height above the bed, and 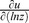 is the slope of the logarithmic velocity profile [37], [38]. We then calibrated motor dial speed to shear stress and used the shear stress values for all analyses (Figure S3).

To compare our measurements of critical shear stress with published values [18], we calculated the non-dimensional Shields stress as 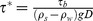 where *ρ_s_* and *ρ_w_* are the densities of the sediment and water respectively, *g* is the acceleration due to gravity and *D* is the diameter of the surface sediments. Shields stress is commonly used to scale between laboratory and field conditions across a wide range of grain sizes [18].

### Statistical analyses

The critical shear stress at which grains first moved was compared across treatments using a general linear mixed model where critical shear stress was a function of the fixed effect of treatment and the random effect of block. Analyses with final caddisfly density included as a covariate (Table S1) suggest that conclusions did not depend on final density (density*treatment: p = 0.1, density: p = 0.31; treatment: p = 0.02), probably due to the relatively narrow range of net densities in the experiment and the controlled number of larvae introduced to the flumes. Thus, density was not included in further statistical modeling. However, because there were differences (Figure S2) in the proportion of the two species in the final count of the polyculture (55% *Arctopsyche*, 45% *Ceratopsyche*), we used a density-weighted average of the critical shear stresses when the species were in monoculture to estimate the additive expectation for incipient motion in the polyculture. Pairwise contrasts corrected for multiple comparisons (multcomp package in R 2.9.0) were used to test for differences between *(i)* the control with no caddisflies and all treatments that did have caddisflies, *(ii)* the two species in monoculture, and *(iii)* the weighted average of the monocultures and the polyculture treatment. Models were fit using the lme4 package in R 2.9.0, and the most parsimonious model was selected by eliminating non-significant terms. To test for an effect of caddisflies on bed stability on shear stresses above critical, we also measured the mass of grains mobilized for each additional increment for which the motor dial speed was increased. We calculated the shear stress when 10–70% of the mass of the surface grains had moved by identifying the shear stresses that bracketed each 20% stage increase, and then used linear interpolation to estimate the shear stress when that given percentage of the grains had moved. This range of shear stress captures bed movement close to incipient motion, as well as substantially above the critical shear stress. A general linear mixed model was used to compare the shear stress required for grains to move between the polyculture and the additive expectation (the weighted average of the monocultures) to test for non-additive effects of the polyculture on the shear stresses beyond critical.

### Net location and habitat shifts

To document the vertical distribution of nets constructed by each species in mono- and polyculture, we ran a parallel behavioral study in which we used plexiglass viewing boxes with sediments layered exactly as in the flume experiment to document where caddisflies were building nets in the benthic substrate. The goal was to determine if species interactions in polyculture lead to a shift in net location and habitat use. This experiment was run as a fully randomized block in which three treatments were established per block: *Ceratopsyche* monoculture, *Arctopsyche* monoculture, and a 50∶50 mix of the two species. One of each of the treatments was randomly assigned to one of three viewing boxes (10 cm long×22 cm wide×20 cm high) during each temporal block. Because the boxes were only as wide as the surface grains, nets were visible across the entire vertical sediment arrangement. The upstream and downstream ends of each plexiglass box were open to allow water to flow through, with 1 mm mesh netting attached to the frame to retain the caddisflies and sediments. We placed caddisflies at a density of 2000 m^−2^ on the surface sediments. After a 1 minute settling period, flow velocity was gradually increased to 30 cm s^−1^. After 24 hours, we identified the species that built each net and used calipers to measure the net depth below the sediment surface. A total of six replicates of each treatment blocked through time were completed. We used kernel density plots to visualize the distribution of caddisfly nets with depth (ggplot2 package in R 2.9.0) and t-tests (stats package in R 2.9.0) to compare mean net depth for individuals of the same species in mono- vs. polyculture.

### Implications for bedload transport in a prototype stream

To assess what the implications of our laboratory experiments might be for sediment mobility in real streams, we used the experimental data to parameterize a common model of sediment motion and applied that model to a prototype stream. The goal of this exercise was not to develop explicit predictions or suggest that the experiments capture the dynamics of sediment mobility in real streams. Rather, the goal was simply to assess whether the multi-species impacts of caddisflies are biologically trivial with respect to physical forces that are represented in bedload models. Or, alternatively, whether the multi-species impacts of caddisflies are sufficiently large that, in principle, they warrant the effort needed to complete more difficult and expensive field tests to detect them.

We started this exercise by determining whether several standard non-dimensional hydrodynamic ratios were comparable between the experimental laboratory flumes and Convict Creek, which we used as a ‘prototype’ stream [34] (Table S2). Convict Creek is a gravel-bedded stream that runs through the Sierra Nevada Aquatic Research Lab (SNARL) where our experimental work was performed, and it is representative of many streams in the eastern Sierra Nevada mountains where both of our focal caddisfly species are naturally present [31]. Comparisons of the non-dimensional hydrodynamic ratios suggest that both lab (flumes) and field (Convict Creek) conditions have a bulk flow that is fully turbulent (flow Reynolds number >2000), that fluid-particle interactions are hydrodynamically ‘rough’ (particle Reynolds number >100), and that empirically derived measures of Shields stress for the control treatment in our flumes (τ^*^_crit__ = _0.034) is consistent with theoretical predictions [39] and field-measured values for loosely-packed, well-sorted gravel particles [18]. Thus, experimental conditions were consistent with the physical forces that would be presumed to be driving and resisting sediment motion in a stream like Convict Creek.

To estimate bedload sediment transport as a function of varying discharge in Convict Creek, we used the Wong and Parker (2006) version of the Meyer-Peter and Muller equation [40] because of its applicability when sediment mixtures are represented by a single characteristic median grain size (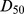). Sediment mass flux is given as 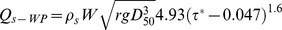 where 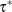 is the non-dimensional shear stress 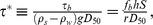 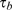 is the shear stress acting on the bed sediments, 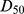 is the median diameter of the bed material, 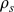 and 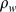 are the densities of the sediment and fluid respectively, *g* is gravitational acceleration, *h* is the flow depth, *S* is the channel slope, *f_b_* is the fraction of the total boundary shear stress available to transport bed sediment, and 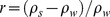. Flow depth as a function of discharge (*Q_w_*) was estimated using Mannings equation 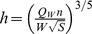 where *W* is the channel width and *n* is the Mannings roughness parameter. From field surveys we obtained *S* = 0.01 and *W = *5.0 m, and from a pebble count of 100 particles determine 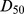 = 22 mm. Based on published values from similar gravel-bedded streams (e.g. Andrews, 2000 [41]), we estimated *n* = 0.04 and *f_b_* = 0.75, and assumed 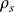 = 2500 kg/m^3^, a typical value for silicate rocks such as the granodiorite of Convict Creek. We used daily discharge data provided by staff of the Sierra Nevada Aquatic Research Laboratory measured at a long-term stream gauge just upstream of our study site. For each of the three scenarios where caddisflies are present, we assumed that τ^*^_crit_ would revert to the control value (0.034) once the threshold of motion shear stress was exceeded. This satisfies the biologically realistic assumptions that the nets holding particles in place will be destroyed when the bed sediment begins to move, that the caddisflies will not rebuild nets until the bed motion stops, and caddisfly individuals will recolonize through drift.

## Results

### Stream mesocosm experiment

**Figure 2:**
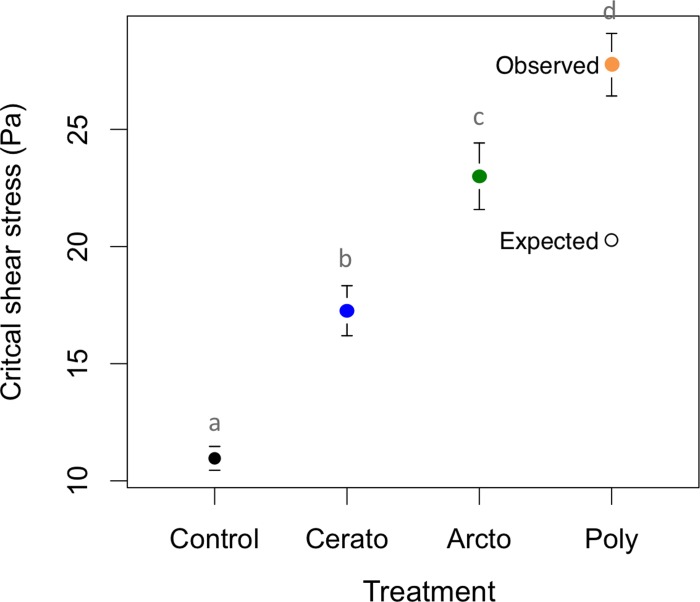
Impacts of monocultures and polycultures of caddisflies on critical shear stress. Values are means ±1 SEM for N = 8. Lowercase letters indicate significant differences between treatments.

Flumes containing single species had significantly higher near-bed shear stress (*τ_b_*) required to initiate sediment motion than did control flumes that were not colonized by caddisfly larvae (Figure 2; Table 1). Compared to control treatments, critical shear stress was 57% higher in treatments with *Ceratopsyche* and 109% higher in treatments with *Arctopsyche* for an average of 83% higher in treatments containing monocultures of caddisflies (Figure 2, p<0.001). Critical shear stress in the patches with the larger species (*Arctopsyche)* was higher than in patches with the smaller species (*Ceratopsyche*), indicating that the larger species had a stronger impact on incipient grain motion. We compared our measurements of shear stress to published values using the non-dimensional shear stress, or Shields stress (Table 1), and found that our measurements, which varied between 0.034–0.086, fall within the range of Shields stress values found in natural gravel-bedded streams [18].

**Table 1:** The threshold of motion for each caddisfly treatment in the experiment.

Treatment	Crtitical Shear Stress (Pa)	Critical Shields Stress	Proportional increaseabove control
Control	11.0±0.5	0.034	0.00
*Ceratopsyche*	17.3±1.1	0.053	0.57
*Arctopsyche*	23.0±1.4	0.071	1.10
Polyculture expected	20.4±0.7	0.063	0.86
Polyculture observed	27.8±1.3	0.086	1.50

The critical shear stress, non-dimensional Shields stress, and proportional increase over the control are given. Critical shears stress values are means ±1 SE. The Shields stress corresponds well to values measured in nature ranging between 0.03–0.08.

If the effect of two species were simply additive, we would expect the critical shear stress in polyculture to be the density-weighted average of the two monocultures or 20.4 Pa. Instead, we found that when the two species were together in a stream, critical shear stress was 27.8 Pa (Table 1, Figure 2), which is greater than the value for either species in monoculture and 26% greater than the additive expectation. Thus, the presence of species in monoculture increased critical shear stress 83% on average above the controls, and the presence of two species in polyculture increased critical shear stress by 150% above the controls.

**Figure 3:**
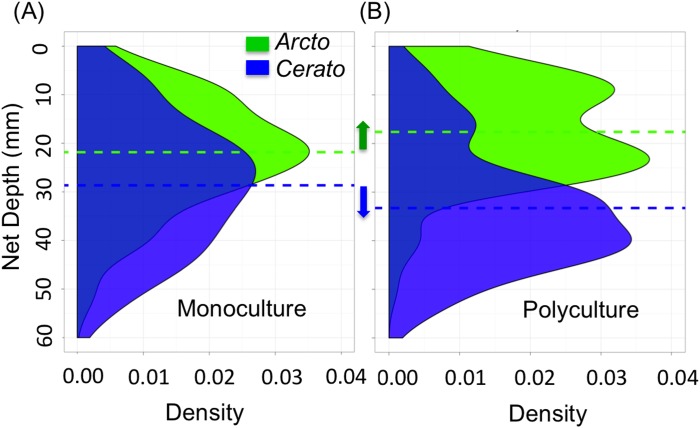
Shifts in net distribution in mono.- vs. polyculture suggest a likely mechanism leading to differences in critical shear stress. (A) A kernel density plot shows the density of nets at a given depth. *Ceratopsyche* (blue) and *Arctopsyche* (green) build at relatively similar depths in monoculture, although the smaller species, *Ceratopsyche*, is capable of building at a significantly deeper depth. Dotted lines represent the mean net depth for each species. (B) When placed together in polyculture, *Arctopsyche* shifts to build its nets at a shallower depth and *Ceratopsyche* shifts to build its nets at a deeper depth.

Our behavioral study of the vertical distributions of caddisfly net locations suggests that the observed non-additive increase in critical shear stress may have resulted from competitive interactions that caused both species to shift their habitat use and net locations when in polyculture. When the two species were in monoculture, *Ceratopsyche* nets were built, on average, at significantly deeper depths than *Arctopsyche* nets, at 29±1.3 mm and 22±1.1 mm (mean ± SEM) below the surface, respectively (Figure 3A, t-test: t = −4.1, p<0.001). When the two species were placed together and forced to interact, both species shifted the depth at which they built nets (Figure 3B). In polyculture, *Arctopsyche* nets were shallower than in monoculture (t-test: t = 2.9, p = 0.004) with a mean net depth of 18±1.0 mm. *Ceratopsyche* nets were deeper than in monoculture (t-test: t = −2.2, p = 0.03) with a mean net depth of 33±1.6 mm.

**Figure 4:**
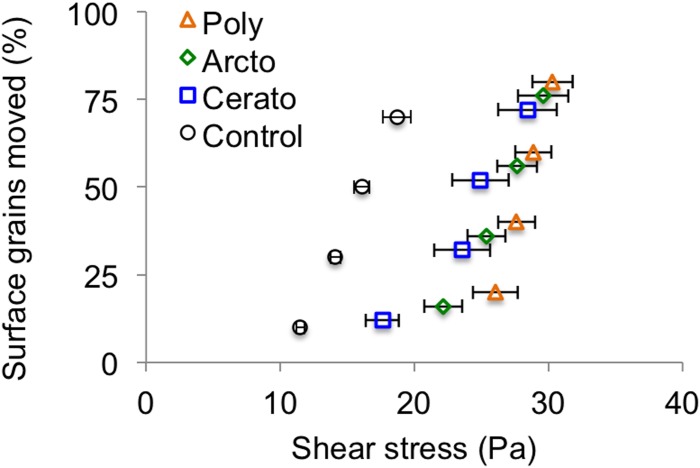
The effect of caddisfly mono.- vs.polycultures on shear stress as the bed eroded beyond the critical shear stress. Values are means of N = 8 replicates ±1 SEM.

The presence of caddisfly nets continued to enhance bed stability even after the shear stress was increased beyond the threshold of motion (Figure 4). We found that the non-additive bed-stabilizing effect of two species in polyculture compared to the additive expectation was significant when the percentage of sediment grains moved was 10% (p<0.001) of the initial mass. But when the shear stress was high enough to move 30% of the grains, the non-additive effect was no longer detectable (p = 0.11).

**Figure 5:**
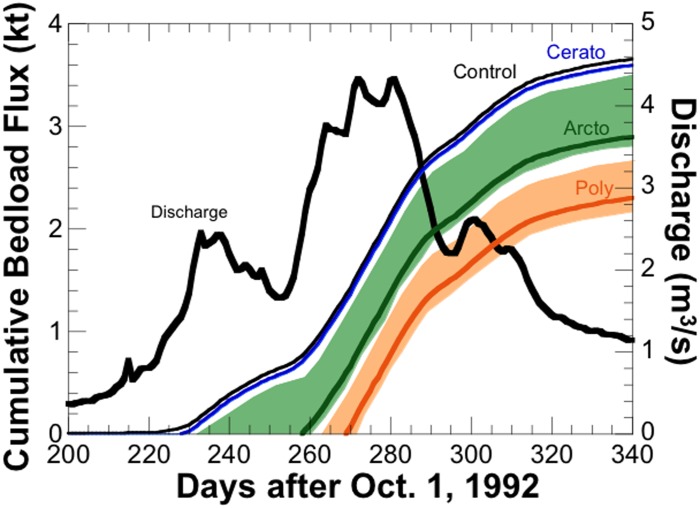
Effects of increasing critical shear stress on predicted bedload sediment transport flux for Convict Creek. The snowmelt hydrograph in Convict Creek for the 1992–1993 water year (which began on October 1, 1992) is represented by the thick black line. The thin black and colored lines represent the predicted cumulative bedload sediment transported for the four values of critical shear stress that correspond to our measurements for the four caddisfly treatments in the experiment: (1) no caddisflies, (2) *Ceratopsyche* monoculture, (3) *Arctopsyche* monoculture, (4) polyculture mixture of both species. Shading represents variation within one standard error.

The snow-melt hydrograph for the 1993 water year in Convict Creek (Figure 5) shows a peak discharge that had a ∼4 year recurrence interval, which can be considered representative of conditions with moderate but substantial bedload sediment transport. Overlain on the hydrograph is the predicted cumulative bedload sediment flux calculated under four scenarios of critical shear stress (τ^*^_crit_) taken from the treatment values measured in the laboratory flume experiment; means are shown as solid lines and shading indicates the range within one standard error. As shown in Figure 5, increasing the critical shear stress would be expected to delay the onset of bed mobility by up to 40 days and reduce the cumulative bedload sediment flux from 3.7 to 2.3 kT for the polyculture scenario (τ^*^_crit = _0.086) over the 140-day snow-melt hydrograph. The sensitivity of sediment flux to increasing critical shear stress is non-linear, with a small effect for the *Ceratopsyche* monoculture (2% reduction), a moderate effect for the *Arctopsyche* monoculture (21% reduction), but a large effect for the polyculture (37% reduction). Again, we caution that Figure 5 represents an extrapolation of results from laboratory flumes to the field and, in doing so, makes many unverified assumptions about the transferability of biological impacts to natural stream conditions. These results should only be viewed as an unverified, *a priori* hypothesis of what impacts caddisflies ‘might’ have in a stream like Convict Creek.

## Discussion

We have demonstrated experimentally that silk nets from a multi-species assemblage of caddisfly larvae have the potential to generate non-additive increases in the critical shear stress required to initiate sediment transport during high flow events compared to single species monocultures. In the sediments of laboratory flumes, the presence of species in monoculture increased critical shear stress 83% on average above the controls, and the presence of two species in polyculture increased critical shear stress by 150% above the controls with no animals. We also found that the strongest effects of polycultures occur at shear stresses close to the threshold of motion but continue to influence sediment mobility at shear stresses beyond critical. These findings build on, and extend, previous studies that have shown caddisfly nets have the potential to increase critical shear stress in streams [22]–[24].

We further used values of critical shear stress measured in our laboratory experiments to parameterize a common model of sediment transport so that we could explore the potential implications of our findings for sediment motion in a natural stream (Convict Creek). Our goal was not to develop explicit predictions, nor to argue that caddisflies impact the dynamics of sediment mobility in real streams. Rather, our goal was simply to assess whether caddisfly nets could affect bedload transport over longer time scales if caddisflies influence critical shear stress similarly to our experimental results. Model results suggest that if the biologically-driven increases in the critical shear stress hold true at field scales, then caddisflies could reduce the magnitude and frequency of bedload flux over annual time scales. Moreover, these impacts would be significantly larger for multi-species assemblages. Taken collectively, our experimental and modeling results suggest that accounting for multiple coexisting species may be an important next step for accurately incorporating biology into quantitative, long-term models of sediment transport and other physical processes. However, we once again caution that this conclusion must be tempered by the fact that the model predictions have yet to be calibrated with field tests, which may include, among other methodologies, transplants to the field of sediments colonized by different caddisfly species in the laboratory, density manipulations using electroshocking or other exclosure techniques, and comparisons across streams that naturally vary in density and the species that are present [42], [43].

The non-additive increase in critical shear stress that we measured in our laboratory flume studies likely resulted from species interactions that drove shifts in habitat use. In polyculture, the different species appeared to partition space within the sediments, with the smaller species *Ceratopsyche* building nets at a relatively deeper depth and the larger species *Arctopsyche* building nets at a relatively shallower depth. We interpret this partitioning of space, which has also been documented in previous studies [27], as evidence for competition among these two species, potentially due to territorial and fighting behaviors [28]. In particular, the increased concentration in near-surface nets built by *Arctopsyche,* combined with the effect of *Ceratopsyche* nets that linked surface grains to subsurface grains, may explain the greater critical shear stress in polyculture.

In monoculture, the larger species *Arctopsyche* had a stronger influence on critical shear stress than the smaller species *Ceratopsyche*. Several possible factors could explain this finding, including differences in *(i)* net size, *(ii)* threads per unit net area, or *(iii)* thread tensile strength [44], [45]. Mechanistically understanding how silk threads stabilize sediment grains is the next experimental challenge, especially under field conditions where silk strength has only been measured for a select few species [45] and is known to vary in strength depending on environmental conditions [46].

Once the bed started to erode in our experiment, we detected a sustained stabilizing effect of caddisfly nets on grain motion, but the non-additive effects of polycultures and the differences between *Arctopsyche* and *Ceratopsyche* monocultures diminished rapidly with increasing shear stress. Although small in magnitude, this reduction in sediment mobility could have significant consequences for annual bedload sediment transport because of the non-linear dependence of bedload flux on excess shear stress [47]; here we suggest three possible explanations for these effects. First, surface grains may require greater shear stress to mobilize because they are held in place by a large number of strong *Arctopsyche* nets, and as those nets break and release individual surface particles, the remaining surface grains are held in place by fewer nets and perhaps rely more on weaker *Ceratopsyche* nets attached from below. Second, surface particles were sometimes mobilized as a clump bound together by nets, which effectively acted as a single larger particle requiring greater stress to lift. In this case, fewer surface and more subsurface nets would need to be broken to release particle clumps, which might diminish the importance of the effect of spatial variation in species-specific net locations across caddisfly composition treatments. Third, as the first surface particles are mobilized, a more irregular bed topography is created, increasing the relative protrusion of remaining particles and enhancing their mobility regardless of caddisfly net composition. Further work is needed to distinguish which combination of the proposed mechanisms is responsible for the effect of diversity on grain movement.

There are a number of inherent limitations in our laboratory experiments that represent opportunities for additional studies. Caddisfly density and diversity vary not only vertically within the surface to subsurface sediments, but longitudinally along riffle habitats in response to velocity, sediment size, and food supply [48]–[50]. This spatial variation could influence incipient sediment motion in a channel through changes in the absolute number of silk nets present, as well as by mediating the size of silk nets and the number and strength of silk threads that caddisflies produce when competition for net building locations is high. Although caddisflies are present year-round, density can also vary between seasons [31], which could limit the times during which caddisflies influence sediment mobility, including the important rain-on-snow floods that occur in winter [51]. In many streams, hydropsychid caddisfly populations also exhibit bi- or multivoltine generations, with distinct shifts in cohorts and thus caddisfly body size, which may lead to temporal variation in the effects caddisflies have on critical shear stress by potentially altering the locations where nets are built and net size since smaller individuals in early instar stages may build smaller nets [30]. The relative abundance of different species, and whether those species are sampled from communities that naturally have one or more species of caddisfly, is likely to be important for future studies if competitive interactions influence where net building sites are chosen, the speed at which nets are built, and the level of aggressiveness displayed by individuals under competitively stressful conditions. Our controlled laboratory study serves as reference condition against which more complex biological interactions should be tested.

In summary, we have used a case study with aquatic insects to demonstrate that a non-additive increase in sediment stability can occur when multiple species of caddisfly are present in a stream. Because our study was conducted in laboratory stream mesocosms, there are inherent limitations to scaling these findings to field conditions. However, to the extent that interacting species cause changes in habitat use in ways that produce non-additive impacts on materials transport, our findings may be representative of how abiotic disturbances such as floods are realized in nature. These results, taken collectively with results from other studies that have explicitly investigated the impact of diversity on abiotic processes [52]–[54], raise the question of whether explicit consideration of species interactions is required to accurately understand how biology influences physical transport processes. As increasing effort and funding is being allocated to linking biology and geomorphology, we suggest our ability to mechanistically incorporate biological impacts into physical models may require that we consider variation among species that co-occur in natural communities.

## Supporting Information

Figure S1Before the experiment, we conducted trials to see how many individuals could colonize the sediment patches that we created in flumes. These trials were conducted over 24 hours, a length of time selected to expedite experimental set up and produce a rough estimate of caddisfly carrying capacity to ensure that we would introduce enough individuals for interactions to occur. For each species, we added an increasing number of individuals to a sediment patch and counted the number of individuals in the patch after 24 hours. (A)We fit the data from the trials to the hyperbolic function 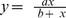 where *y* is the number of individuals that settled in the patch, *a* is the maximum number of individuals predicted to colonize a patch (a.k.a. carrying capacity, *K*), *b* is the inflection point of the curve, and *x* is the number of individuals initially introduced to the patch. This allowed us to estimate parameters *a* and *b* for each species - which represent maximum density (*K*), and the half saturation constant, respectively. We then estimated *x*, the number of individuals we would initially need to introduce to a patch, for a given percentage of *K*. Given constraints on our ability to collect so many individuals for each successive block of the experiment, we chose 138 individuals, which represented 40% of *a* for *Ceratopsyche* and 59% of *a* for *Arctopsyche* according to this 24 hour trial. Given that we counted fewer individuals than this in the actual patch (see Figure S2) during the experiment, this suggests that individuals were in fact forced to interact and compete over limited space. (B) We used t-tests to compare the body size of individuals from the two species used in our experiments. The extended (i.e. uncurled) length of an *Arctopsyche* individual (n = 200) was significantly longer (t = 43.8, p<0.001) than *Ceratopsyche*. *Arctopsyche* individuals (n = 3) were also significantly heavier (t = 4.6, p = 0.04) than *Ceratopsyche* individuals. Differences in body size are key factors that might regulate the quantity and depth of nets for each species.(TIF)

Figure S2 (A) The number of individuals recovered from the experimental patches during the simulated flood shows that a significantly (F_2,21_ = 7.03, p<0.01) larger number of individuals were counted in the *Ceratopsyche* vs. *Arctopsyche* patches. The number of settlers in the polyculture was not different from the monoculture *Arctopsyche* (F_2,21_ = 7.03, p = 0.12) or *Ceratopsyche* (F_2,21_ = 7.03, p = 0.13) patches. When the two monocultures were averaged, the number of settlers in monoculture was not significantly different from the number of settlers in polyculture (t = −0.29, p = 0.77). Although we initially seeded the polyculture patches with a 50∶50 mix of the two species, the final settling mixture was, on average, composed of 55% *Arctopsyche* and 45% *Ceratopsyche* individuals. As such, we used a density-weighted average to estimate the additive expectation for the polyculture (see Figure 2). (B) The total caddisfly biomass in the various treatments. ANOVA with Tukey posthoc tests revealed that biomass was not different between the mix polyculture and *Arctopsyche* monoculture (p = 0.11), but both the polyculture (p<0.001) and *Arctopsyche* monoculture (p<0.001) were higher in biomass than the *Ceratopsyche* monoculture. The average of the monocultures was not different from the polyculture (t = 1.7, p = 0.12), which is largely driven by the biomass of *Arctopsyche*. For both panels, circles represent the full mean ±1 SEM for each of the 8 replicates of the experiment. Triangles in the polyculture treatment represent the number or biomass of the two different species separately. Black symbols represent *Arctopsyche* and gray symbols represent *Ceratopsyche*.
(TIF)

Figure S3 Prior to the experiment, we calibrated motor dial position to near-bed shear stress using velocity profiles. These measurements were taken before the experiment because it was not possible to measure velocity during the experiment itself when the caddisflies and their nets were present. We measured velocity profiles above the center of the working rock patch using an arrangement of surface rocks cemented to plexiglass at a height and location identical to rocks in the experiment. (A) Examples of velocity profiles for two motor dial positions. To estimate shear stress from the velocity profiles, we used standard methods to find the slope of the linear regression of velocity on the log of depth in the measured profile (see Methods). (B) Shear stress was calculated for motor dial positions 20, 30, 40, and 50, and linear regression was used to estimate the relationship between motor dial position and shear stress. Dial speed positions documented in the experiment were converted to shear stress values using the equation shown in the bottom right of panel B.
(TIF)

Table S1 Net density has the potential to play a significant role in influencing the threshold of sediment motion [22]. We did find that there were differences in the number of caddisfly settlers, which may translate to differences in net density, between the monoculture treatments of *Arctopsyche* and *Ceratopsyche* (Figure S2). In spite of this, we found little evidence that differences in final density across species treatments influenced critical shear stress in the experiment. We compared two mixed effects models, where the full model included critical shear stress in the monocultures as a function of the fixed effects of caddisfly species treatment, caddisfly density, and their interaction and the random effect of block. After finding that the interaction term and the density term were non-significant, we ran a reduced model that included the fixed effect of treatment and the random effect of block. Model selection criteria indicate that the full model does not explain any more variation in the data than the reduced model, suggesting that differences in caddisfly density across treatments had minimal impact on the threshold of sediment motion in our experiment.
(XLSX)

Table S2 Comparison of the estimated hydraulic variables of the flumes under conditions for incipient motion of the control (no caddisflies) and polyculture treatments to nearby Convict Creek, CA.
(XLSX)
