# Supplementary figures and images for: Non-Additive Increases in Sediment Stability Are Generated by Macroinvertebrate Species Interactions in Laboratory Streams
Source: PLoS One. 2014 Aug 7;9(8):e103417. doi: 10.1371/journal.pone.0103417 (PMC4125303; doi:10.1371/journal.pone.0103417)

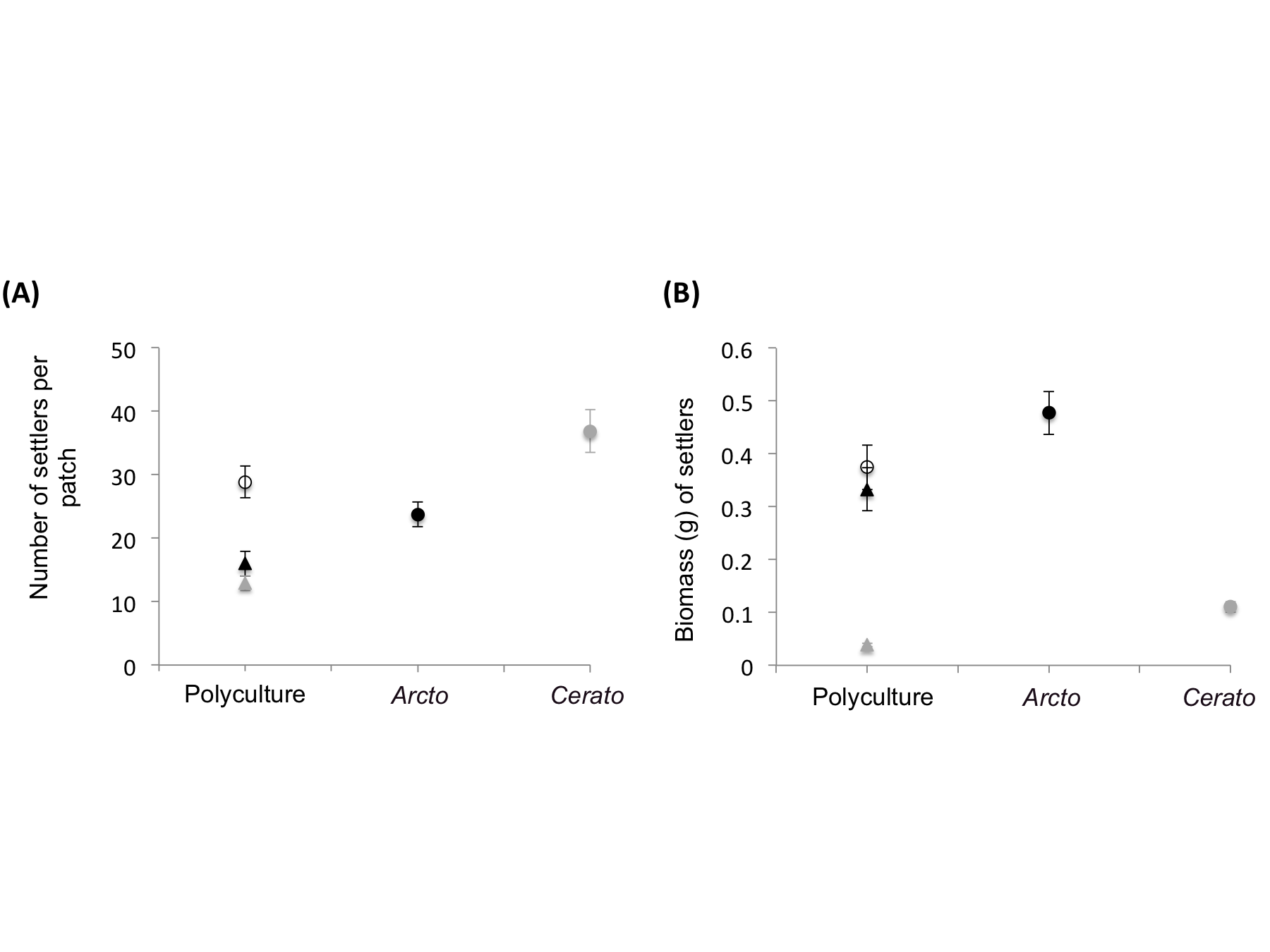

Supplement: Figure S2 — (A) The number of individuals recovered from the experimental patches during the simulated flood shows that a significantly (F2,21 = 7.03, p<0.01) larger number of individuals were counted in the Ceratopsyche vs. Arctopsyche patches. The number of settlers in the polyculture was not different from the monoculture Arctopsyche (F2,21 = 7.03, p = 0.12) or Ceratopsyche (F2,21 = 7.03, p = 0.13) patches. When the two monocultures were averaged, the number of settlers in monoculture was not significantly different from the number of settlers in polyculture (t = −0.29, p = 0.77). Although we initially seeded the polyculture patches with a 50∶50 mix of the two species, the final settling mixture was, on average, composed of 55% Arctopsyche and 45% Ceratopsyche individuals. As such, we used a density-weighted average to estimate the additive expectation for the polyculture (see Figure 2). (B) The total caddisfly biomass in the various treatments. ANOVA with Tukey posthoc tests revealed that biomass was not different between the mix polyculture and Arctopsyche monoculture (p = 0.11), but both the polyculture (p<0.001) and Arctopsyche monoculture (p<0.001) were higher in biomass than the Ceratopsyche monoculture. The average of the monocultures was not different from the polyculture (t = 1.7, p = 0.12), which is largely driven by the biomass of Arctopsyche. For both panels, circles represent the full mean ±1 SEM for each of the 8 replicates of the experiment. Triangles in the polyculture treatment represent the number or biomass of the two different species separately. Black symbols represent Arctopsyche and gray symbols represent Ceratopsyche. (TIF) [file pone.0103417.s002.tif]
